# Causes of poor eye contact in infants: a population-based study

**DOI:** 10.1186/s12886-021-02151-7

**Published:** 2021-11-07

**Authors:** Mette Levinsen, Malene Landbo Børresen, Laura Roos, Karen Grønskov, Line Kessel

**Affiliations:** 1 Department of Ophthalmology, Rigshospitalet, Valdemar Hansens Vej 13, 2600 Glostrup, Denmark; 2 Department of Pediatrics and Adolescent Medicine, Rigshospitalet, Copenhagen, Denmark; 3 Department of Clinical Genetics, Rigshospitalet, Copenhagen, Denmark; 4 Department of Clinical Medicine, University of Copenhagen, Copenhagen, Denmark

**Keywords:** Infant, Delayed visual maturation, Infants, Nystagmus, Poor eye contact, Vision

## Abstract

**Background:**

Establishing eye contact between infants and parents is important for early parent-child bonding and lack of eye contact may be a sign of severe underlying disease. The aim of the study was to evaluate the causes of poor or lacking eye contact in infants.

**Methods:**

Cross-sectional study reviewing all referrals of infants ≤1 year of age from January 1rst, 2016 to December 31rst, 2018. Medical information was retrieved from patient files covering pregnancy, birth, diagnostic work-up and ocular parameters such as refraction, visual acuity and structural findings.

**Results:**

We identified 99 infants with poor or lacking eye contact. The relative frequency of causes was neurologic disease 36.4% (36/99), delayed visual maturation 24.2% (24/99), ocular disease 21.2% (21/99) and idiopathic infantile nystagmus 4.0% (4/99). Fourteen infants had a visual function within age-related norms at first examination despite poor eye contact at the time of referral. Of the infants with available data, 18/27 (33.3%) with neurologic cause, 15/23 (65.2%) with delayed visual maturation and 9/21 (42.9%) with ocular cause had visual acuity within the age-related norm at latest follow-up (0-41 months). In 23 infants, a genetic cause was found.

**Conclusion:**

Poor eye contact in infants may be a sign of severe underlying disease, such as neurological or ocular disease. Close collaboration between pediatric ophthalmologists and neuro-pediatricians are warranted in the management of these infants.

**Supplementary Information:**

The online version contains supplementary material available at 10.1186/s12886-021-02151-7.

## Background

Humans are born with the ability to see but the visual system of a newborn needs to undergo dramatic changes for the child to develop adult visual functions. Normal visual function and visual development requires structurally normal eyes and optic radiations as well as a normally functioning brain to process visual input. Eye contact is an important step for early parent-child interaction and lack of eye contact or apparent visual behavior should alert parents and clinicians.

The eye of a newborn undergoes significant structural changes. The anterior segment of the eye bends incoming rays of light to ensure that images are formed on the retina. These refractive components change significantly throughout childhood but mainly in the first year of life with decreasing power of both cornea and lens as the axial length elongates [1, 2]. The retinal fovea is responsible for visual acuity and the foveal development with centrifugal displacement of inner retinal layers begins in utero and is complete by adolescence [3]. Visual input from the eyes is transmitted via the optic nerves to form synapses in the lateral geniculate nucleus (LGN), from which the optic radiations extend to the primary visual cortex in the occipital lobes. These central projections also undergo significant changes in early infancy and throughout childhood. The fibers of the optic nerve become myelinated within the first year of life [4] and the optic nerve and optic nerve sheath increases in diameter in the first four years of life [5]. The optic radiations are not myelinated at birth and visual development follows the rate of myelination [6, 7], which is complete at 40 months of age [8]. The primary visual cortex (V1) is the first cortical area that processes visual information and V1 undergoes synaptic and dendritic refinement to reach adult appearance at around 2 years of age [9].

It has been suggested to classify infants with poor visual contact into three groups depending on the etiology: 1) “normal” infants with/without idiopathic infantile nystagmus, including infants with isolated delayed visual maturation, 2) infants with neurodevelopmental delay and 3) infants with obvious structural ocular abnormalities including albinism [10]. Delayed visual maturation is defined as a normal infant with a transient visual deficit. Although visual outcome is expected to be good, it has been reported that those infants may later demonstrate poor neurological outcome such as learning disabilities, attention deficit disorders or other neurological diseases at long term follow-up [11]. Absent or poor eye contact may be a sign of severe underlying disease. To the best of our knowledge, there are no previous studies on the frequency of underlying causes of poor eye contact among infants. The objective of this study was to evaluate the causes of poor or lacking eye contact among infants below one year of age.

## Methods

The study was a retrospective review of hospital records. All referrals to the Department of Ophthalmology, Rigshospitalet on infants < 1 year of age at the time of referral from January 1rst, 2016 to December 31rst, 2018 were reviewed. Infants referred due to suspicion of poor eye contact and infants with poor eye contact at the time of first consultation were included in the study. Only infants living in the Capital Region of Denmark were included. The Capital Region covers one third of the Danish population and is served by one eye department.

Medical files were reviewed and ophthalmological and pediatric information was retrieved. Moreover, data concerning neuroimaging, metabolic screening, genetic testing (see Additional file 1 for further details) and electrophysiologic testing were obtained from patient files. Electrophysiologic testing included contact- or skin-electrode electroretinograms (ERG) and flash-visual evoked potentials (VEP) or albino-VEP. The causes of poor eye contact were grouped into three groups depending on the etiology [10]: 1) infants with idiopathic infantile nystagmus (group 1a) and infants with isolated delayed visual maturation (group 1b), 2) infants with neurodevelopmental delay and 3) infants with structural ocular abnormalities.

Visual acuity was evaluated at the latest available time point and the method of visual acuity measurement was recorded. Methods of measurement included preferential looking measurements such as Teller Acuity Cards and Cardiff Pictures and recognition acuity tests such as Kay Pictures and Snellen Acuity Chart. Visual acuities were compared with the age-related normative values reported by Leone et al. [12]. They reported mean binocular VA and standard deviation given in cycles/degrees based on Teller acuity cards. For infants born preterm, the age-related value was based on expected date of delivery. For refractive measurements, only cycloplegic values were recorded and values were reported in spherical equivalents (SEQ) by subtracting half of the negative cylinder value from the spherical value in Dioptres (D). We calculated the prevalence of poor visual function during the first year of life as total number of infants with poor eye contact < 1 year of age divided by total number of births in the capture area. Non-normally distributed variables are summarized as medians and range. Categorial variables are summarized as frequencies and proportions. The difference between continuous variables were examined by the Mann–Whitney *U* test. A 2-sided *p*-value < 0.05 was considered statistically significant.

## Results

From January 1rst, 2016 to December 31rst, 2018, 802 infants ≤1 year of age were referred to the Department of Ophthalmology at Rigshospitalet. Of the 802 infants, 122 infants (15.2%) were referred due to lack of eye contact or were found to have poor eye contact at the time of first consultation. Twenty-three infants not residing in the Capital Region of Denmark at the time of referral were excluded, leaving 99 infants (53 boys/46 girls) included in the present study. Clinical characteristics of the study population are provided in Table 1.

**Table 1:** Baseline demographics of infants with poor or lacking eye contact referred to the Department of Ophthalmology, Rigshospitalet

	Normal vision at first consultation	Group 1a – idiopathic infantile nystagmus	Group 1b – isolated delayed visual maturation	Group 2 – neurodevelopmental delay	Group 3 – structural ocular abnormality
Number of infants (boys/girls)	14 (7/7)	4 (2/2)	24 (14/10)	36 (19/17)	21 (14/7)
Gestational age (days, median (range))^a^	281 (243-292)	271 (273-291)	271 (197-292)	275 (203-297)	280 (204-294)
Very preterm (28 – 31 GA, n (%))	0 (0%)	0 (0%)	2 (8.3%)	1 (2.8%)	1 (5.0%)
Moderate to late preterm (32 - 37 GA, n (%))	3 (21.4%)	0 (0%)	6 (25.0%)	8 (22.2%)	2 (10.0%)
Born at term (> 38 GA, n (%))	11 (78.6%)	4 (100.0%)	16 (66.7%)	27 (75.0%)	17 (81.0%)
Pregnancy, eventful/total (n)	4/14	0/4	8/24	20/36	4/21
Birth, eventful/total (n)	3/14	1/4	7/24	19/36	6/21
Age at referral (weeks), median (range)	12.4 (6.1-32.1)	10.0 (9.1-10.4)	12.0 (5.6-34.7)	20.4 (0.3-45.0)	14.7 (4.9-34.7)
Follow-up (months), median (range)	0 (0-5.5)	14.4 (9.6-39.0)	5.4 (2.0-20.2)	17.0 (2.0-35.9)	16.5 (1.0-41.1)
Referring party, n (%)					
Pediatrician	4 (28.6%)	2 (50.0%)	14 (58.3%)	30 (83.3%)	10 (47.6%)
Centre for rare diseases	0 (0%)	0 (0%)	0 (0%)	4 (11.1%)	2 (9.5%)
Ophthalmologist	5 (35.7%)	2 (50.0%)	5 (20.8%)	2 (5.6%)	4 (19.0%)
General practitioner	5 (35.7%)	0 (0%)	5 (20.8%)	0 (0%)	5 (23.8%)
Diagnostic work-up					
Last VA (logMAR), median (range)^b^	0.88 (1.84-0.0)	0.73 (1.68-0.22)	0.62 (1.5-0.0)	0.62 (1.3-0.10)	0.62 (NLP-0.10)
Refraction (Diopter), median (range)^c^	2.8 (−0.4-3.9)	2.8 (−0.4-4.4)	1.9 (−1.3-4.4)	1.6 (−7.1-8.4)	2.4 (−6.8-10.0)
Brain imaging, n/total (%)^d^	4/14 (28.6%)	3/4 (75.0%)	6/24 (25.0%)	30/36 (83.3%)	9/21 (42.9%)
MRI, n/total (%)	2/14 (14.3%)	3/4 (75.0%)	2/24 (8.3%)	27/36 (75.0%)	8/21 (38.1%)
CT, n/total (%)	1/14 (7.1%)	0/4 (0%)	0/24 (0%)	6/36 (16.7%)	0/21 (0%)
US, n/total (%)	2/14 (14.3%)	0/4 (0%)	4/24 (16.7%)	11/36 (30.6%)	2/21 (9.5%)
Abnormal brain imaging, n/total (%)	2/4 (50.0%)	0/3 (0%)	2/6 (33.3%)	26/30 (86.7%)	5/9 (55.5%)
Urinary metabolic screen, n/total (%)	1/14 (7.1%)	0/4 (0%)	3/24 (12.5%)	22/36 (61.1%)	8/21 (38.1%)
Genetic testing, n/total (%)	1/14 (7.1%)	3/4 (75.0%)	3/24 (12.5%)	26/36 (75.0%)	12/21 (57.1%)
Genetic diagnosis, n/total (%)	0/1 (0%)	0/3 (0%)	0/3 (0%)	16/26 (61.5%)	7/12 (58.3%)
Ocular electrophysiology, n/total (%)	0/14 (0%)	4/4 (100%)	0/24 (0%)	5/36 (13.9%)	7/21 (33.3%)

^a^Information about gestational age at birth was unavailable for one infant in Group 3

^b^Methods of measurement were Teller Acuity Cards (*n* = 53), Cardiff Pictures (*n* = 17), Kay Pictures (n = 5), Snellen Acuity Chart (n = 4) and unknown (*n* = 6). Visual acuity was measured without correction (*n* = 40), with correction (*n* = 16) and unspecified (*n* = 29). Visual acuity measurements were unavailable for four infants presenting with normal visual function, one infant with delayed visual maturation and nine infants in Group 3

^c^Refraction is presented as spherical equivalent for right eyes

^d^For brain imaging, the subtotals of infants undergoing MRI, CT and US do not always add up to the total number of infants in each group as one infant could undergo more than one type of brain imaging

*CT* computer tomography, *ERG* electroretinogram, *GA* gestational age in weeks, *logMAR* logarithm to the minimal angle of resolution, *MRI* magnetic resonance imaging, *NGS* next generation sequencing, *NLP* no light perception, *US* ultrasound, *VA* visual acuity, *VEP* visually evoked potential

Pregnancy was uneventful in 62.1% (59/95 infants with available information on birth). Problems encountered during pregnancy ranged from gestational diabetes mellitus to severe structural malformations detected in the fetus. The median gestational age (GA) at birth was 39 + 4 weeks (minimum and maximum GA; 28 + 1 and 42 + 3 weeks respectively). Four infants were born very preterm (28 to 31 weeks) of which one infant had periventricular leukomalacia (PVL), one infant had intraventricular hemorrhage without PVL and one had temporary stage 2 retinopathy of prematurity. Nineteen infants were born moderate to late preterm (32 to 37 weeks) of which eight infants with neurodevelopmental delay had ischemia in occipital and parietal lobes and six infants had other abnormal findings on brain imaging. Median age at referral was 13.4 weeks (range 0.3-45.0). However, infants with neurodevelopmental delay were significantly older than all other infants at the time of referral (P for age at referral in groups 1 + 3 versus group 2 = 0.004).

**Fig. 1:**
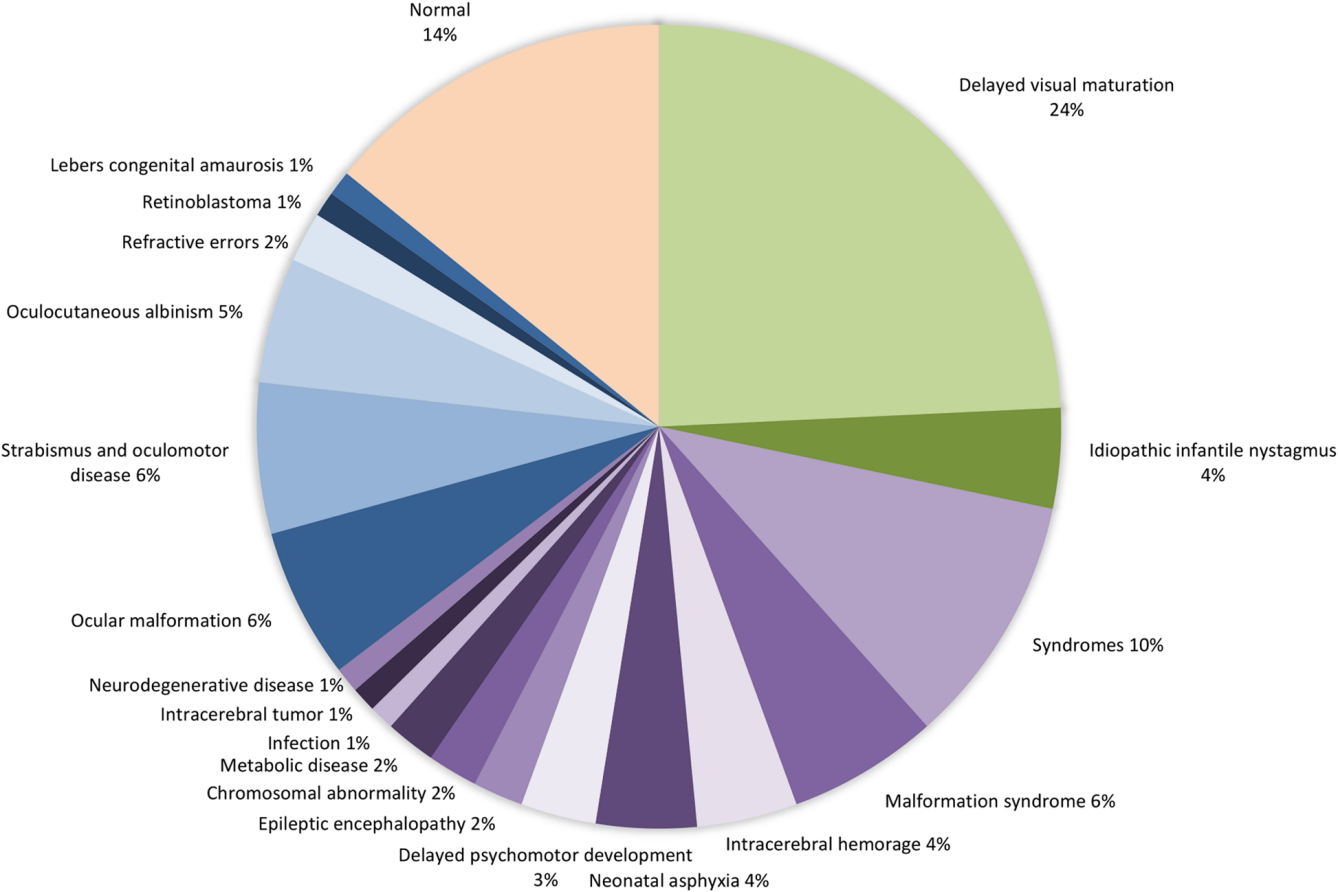
Diagnostic findings in infants with poor eye contact. Diagnostic findings for “normal” infants with/without idiopathic infantile nystagmus, including infants with isolated delayed visual maturation (group 1) are shown in green. Diagnostic findings for infants with a neurological abnormality (group 2) are shown in purple. Diagnostic findings for infants with an obvious structural ocular cause of poor vision (group 3) are shown in blue. Infants who were referred with a suspicion of poor vision but were found to have normal visual function at first examination are shown in yellow. None of the infants with idiopathic infantile nystagmus had structural ocular abnormalities

In 14 infants (14.1%) eye contact and vision was found to be within the age-related range at first presentation to the eye department (Table 1). For the remaining infants, the relative frequency of causes of poor eye contact was idiopathic infantile nystagmus in 4.0% (4/99), delayed visual maturation in 24.2% (24/99), neurodevelopmental delay in 36.4% (36/99) and ocular disease in 21.2% (21/99). Among infants with ocular disease, 33.3% (7/21) had associated nystagmus. Diagnostic findings in infants with poor eye contact are presented in Fig. 1.

**Fig. 2:**
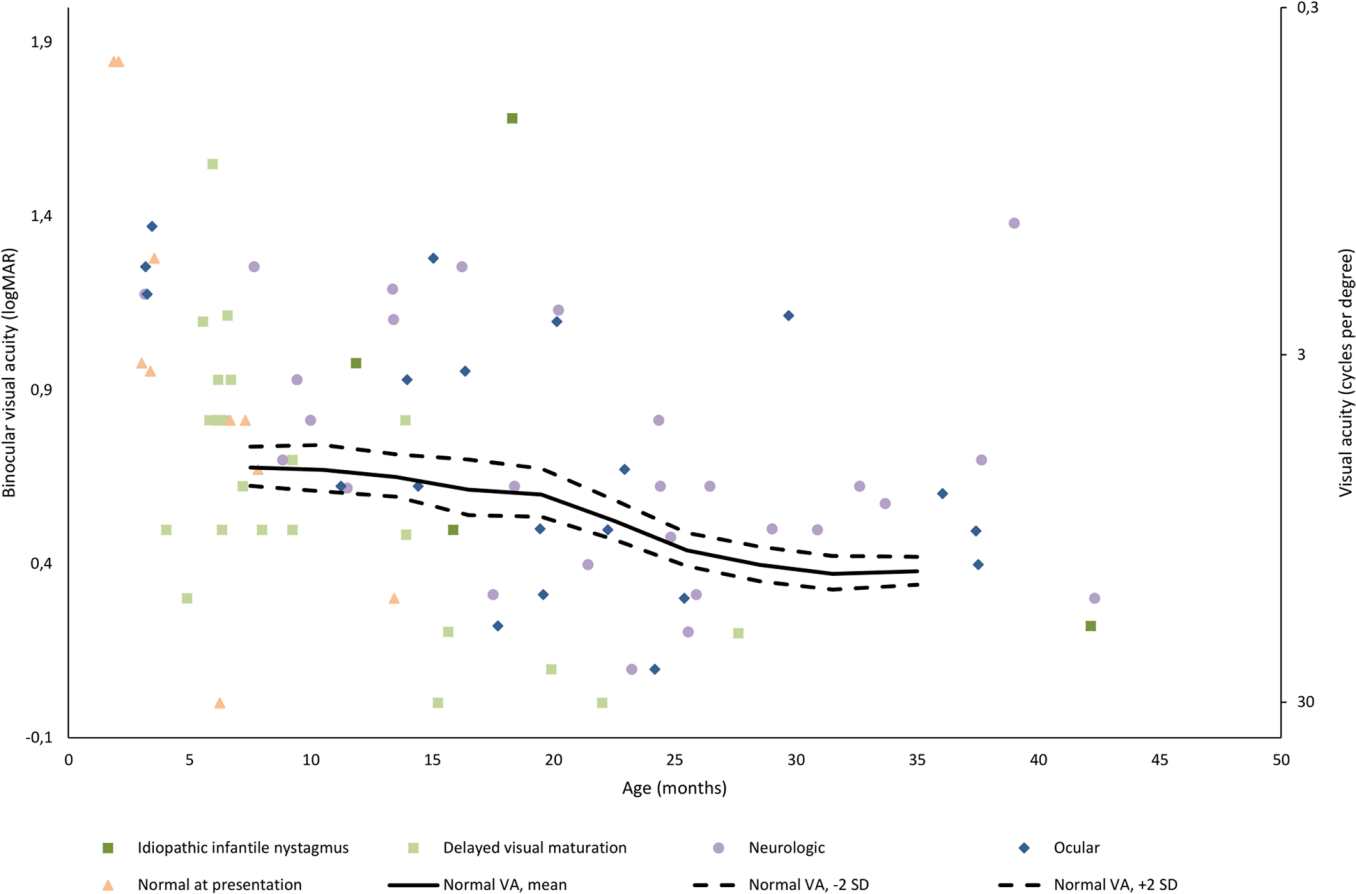
Binocular visual acuity at the latest available follow-up. The normal range for visual acuity (VA) tested by Teller acuity charts was adapted from Leone et al. 2014. Visual acuity measurements were unavailable for four infants presenting with normal visual function, one infant with delayed visual maturation and nine infants with neurological diseases

Of the infants with available data, the visual acuity was within the age-related norm at latest follow-up (0-41 months) for 2/4 (50.0%) with idiopathic infantile nystagmus, 18/27 (33.3%) with neurologic cause, 15/23 (65.2%) with delayed visual maturation and 9/21 (42.9%) with ocular cause (Fig. 2).

Strabismus was present among 18.2% infants (18/99) and amblyopia was reported in 17.2% of cases (17/99). One third of the infants (37/99, 37.4%) had delayed psychomotor development, of which 33 infants had a neurological abnormality.

Overall, neuroimaging was performed in 52 infants (52.5%) of which 35 had abnormal findings (67.3% of those tested, Table 1). Brain imaging was performed in 30 of the 36 infants with neurodevelopmental delay of which 26 had an abnormal scan. The morphological findings were ischemia in occipital and parietal lobe (*n* = 8), delayed myelination in optic radiations (*n* = 3), PVL (*n* = 2), optic atrophy/hypoplasia (n = 2) and other abnormal findings (*n* = 11).

Nearly half of the infants (45/99, 45.5%) underwent one or more genetic test which resulted in a diagnosis in 51.1% (23/45) of infants tested, see Additional file 2. Among infants with neurodevelopmental delay, syndromes (n = 11) were the most common diagnosis. Panels based on next generation sequencing (NGS) had the highest diagnostic yield (14/23, 60.9%), followed by comparative genomic hybridization/array-comparative genomic hybridization (CGH) (5/23, 21.7%), whole exome sequencing (3/23, 13.0%) and chromosome analysis (2/23, 8.7%). Metabolic screening was performed in 34 infants (34.3%) and was abnormal in two infants. One infant with oculomotor apraxia had increased excretion of methylmalonacid corresponding to vitamin B12 deficiency. One infant had increased urinary secretion of sulfocystine and taurine in combination with decreased plasma levels of cystine corresponding to a diagnosis of Molybden co-factor synthesis deficiency.

Overall, 16 infants underwent electrophysiology testing including VEP and ERG. The infants were between three and 20 months old (median 7 months) when electrophysiology was performed (Additional file 3). Three infants with neurologic cause of poor vision had abnormal VEP, two infants with oculocutaneous albinism had misrouting of the optic pathways and one infant was found to have Lebers congenital amaurosis with a non-recordable ERG.

Of the included 99 infants, 85 infants had impaired eye contact at first consultation. From January 1rst, 2016 to December 31th, 2018, 66,789 infants were born in the Capital Region of Denmark (Statistics Denmark, accessed on March 23, 2020, https://www.statistikbanken.dk/10017), hence 12,7 infants per 10,000 liveborn were found to have poor visual function during the first year of life.

## Discussion

We evaluated a group of infants referred to an eye department because of or with poor visual function in the first year of life and found that the most common cause of poor vision in infancy was related to neurological abnormalities followed by delayed visual maturation, structural ocular abnormalities and idiopathic infantile nystagmus. A relatively large proportion, 14.1%, were referred with suspicion of poor vision but were found to have normal eye contact and normal age-related visual function at the time of examination. Infants with neurological abnormalities were older at the time of referral to the eye department. This may reflect that their neurological disease required work-up at a pediatric department before they were referred for an eye examination.

In a three-year period, we saw 99 infants referred because of poor visual function. The infants were born in the Capital Region of Denmark, which covers one third of the Danish population and is served by one department of ophthalmology. Thus, all infants referred with poor visual function was captured by this study. A large number of the infants had irreversible causes of poor vision, but some reached the age-related normative values within the follow-up period showing that parents can expect some visual development in an infant with poor eye contact. For infants with a neurologic or ocular cause, the visual acuity was generally below age-related norms at latest follow-up. Others have found that most infants catch up with visual function but that the catch-up is slower in those infants with mental retardation and neurological issues [13, 14]. In our study, strabismus and amblyopia were frequent findings as they were present among 18.2 and 17.2% of the infants. In comparison, a previous study has found the prevalence of strabismus and amblyopia in healthy children in Sweden to be 3.1 and 2.9%, respectively [15].

Delayed visual maturation is a diagnosis that requires other causes of poor visual function to be ruled out. For infants with delayed visual maturation, the majority had visual acuity within or better than the age-related norm based on preferential looking charts. This is to be expected given the diagnosis and based on other studies [16]. One-third of the infants with delayed visual maturation was born preterm. Visually evoked potentials are known to be affected in preterm infants although they tend to overcome the immaturities within the first year of life [17] suggesting that the immaturity of the brain is causing delayed visual maturation in some cases. Infants with delayed visual maturation may, however, have normal visually evoked potentials and electroretinographies in spite of the apparent lack of visual behavior [18]. Although the visual outcome is expected to be good in infants with delayed visual maturation, some may show neurological deficits later in life [11, 19]. Because of the recent inclusion period in our study, we were not able to provide long-term follow-up of the infants with delayed visual maturation.

A great concern to parents of infants with poor or lacking eye contact is often that the lack of eye contact is a sign of autism. It has been reported that attention to eyes is reduced in infants who are later diagnosed with autism-spectrum disorder [20], whereas others have found that infants who later develop autism-spectrum disorder have normal face-orientation [21]. Vision is important for social interaction and infants with visual impairment have been reported to be more likely to exhibit autism-associated behavioral traits [22] and autism-spectrum disorders are not rare findings among visually impaired infants [23]. Although this study could not provide long-term follow-up, it is important to emphasize that none of the infants in this study had an autism-spectrum disorder diagnosis, although some had severe neurological disease that may be associated with autistic features.

We evaluated the degree of diagnostic work-up to help health care personnel decide how best to perform the investigative work-up among infants with poor eye contact. Genetic analyses were performed in half of the infants and targeted NGS panels showed the highest diagnostic yield. It is important to keep in mind that targeted NGS panels were only used when diagnostic considerations could be narrowed down to possibilities covered by a panel. Some tests were used more broadly as screening, e.g. array-CGH, or were only employed when a genetic cause was suspected but had failed to be revealed using standard genetic work-up, e.g. whole exome sequencing when targeted NGS panels did not find a genetic cause. The capture of inborn errors of metabolism (IEM) by urine metabolic screening was very low. This reflected the low incidence of IEM and in particular the low incidence of organic acidurias, aminoacidopathies and mucopolysaccharidoses, which are the IEM that can be captured by urine metabolic screening. Diseases within the range of IEM consist of more than 1000 diseases with a very low incidence of each of the diseases but a total incidence of 1: 1000-2000 births [24]. Ocular manifestations occur in a number of metabolic diseases and an ophthalmological examination may in some cases reveal the diagnosis [25]. In addition, a urine metabolic examination in the infant with poor eye contact with unknown cause is essential as treatment might be possible [26].

The present study was a cross-sectional study based on retrospective review of hospital records. All cross-sectional studies have some limitations. In this study, the main limitation was that not all infants underwent the same diagnostic work-up. Thus, some findings may have been missed. It can be debated whether it is ethically justifiable to subject all infants to a rigorous testing set-up where some examinations, e.g. brain MRI, may require general anesthesia. Moreover, our visual acuity data was compared to a normative data set from Leone et al. [12] based on Teller acuity cards. Our study included a range of vision tests making the data less comparable. However, 63.4% (53/85) of visual acuity measurements in our study were based on Teller acuity, the majority of tests (88.2%; 75/85) were based on uncrowded tests and all infants had visual acuity measured binocularly as in the study by Leone. Our study is unique in that it covered a large proportion of a whole nation (1/3 of the Danish population lives in our uptake area) and that all medical information were accessible to us. Our findings are applicable to other nations with similar socio-economic background. Nations with a markedly different society, e.g. developing nations with high prevalence of neonatal infections, are likely to have different causes of poor vision in infants.

## Conclusion

It is not uncommon for parents and health care personnel to suspect inadequate visual function in infants. In this study we provide a detailed characterization of the different causes of poor eye contact in infants. Neurodevelopmental delay was found to be the leading cause of poor eye contact (36.4%) followed by delayed visual maturation (24.2%) and ocular disease (21.2%). A large proportion of the infants are expected to develop normal visual acuity later in life but those with an ocular or neurologic finding often have irreversible causes of poor vision. When health care personnel encounter infants with poor visual attention it seems advisable to include both ophthalmologists and pediatric neurologists in assessing the cause. In absence of an obvious cause, the infant may need to undergo a number of investigations including brain MRI, electrophysiology, metabolic screening and genetic work-up to determine the cause of poor visual attention. As the proportion of infants with delayed visual maturation is quite high, it seems acceptable to await the natural development for some months if the infant is thriving without signs of neurological disease. However, later presentation of underlying neurological disease should be kept in mind.

## Supplementary Information

**Additional file 1.** Detailed description of methods for genetic testing. Detailed description of methods for karyotyping, chromosomal microarray, exome sequencing and next generation sequencing.

**Additional file 2.** Genetic findings in infants with poor eye contact. Detailed description of genetic findings among infants with poor eye contact.

**Additional file 3.** Electrophysiology testing including visual evoked potentials and electrode electroretinograms. Detailed description of electrophysiology testing among infants with poor eye contact.

## Data Availability

All data generated or analysed during this study are included in this published article and its supplementary information files.

## Abbreviations

CGH: Comparative genomic hybridization
CT: Computer tomography
D: Dioptres
ERG: Electrode electroretinograms
GA: Gestational age
IEM: Inborn errors of metabolism
logMAR: Logarithm to the minimal angle of resolution
MRI: Magnetic resonance imaging
NGS: Next generation sequencing
NLP: No light perception
PVL: Periventricular leukomalacia
SEQ: Spherical equivalents
US: Ultrasound
V1: Primary visual cortex
VA: Visual acuity
VEP: Visual evoked potentials
