# Supplementary material for: Causes of poor eye contact in infants: a population-based study
Source: BMC Ophthalmol. 2021 Nov 7;21:388. doi: 10.1186/s12886-021-02151-7 (PMC8572507; doi:10.1186/s12886-021-02151-7)
Supplement: Supplementary file 1 — Additional file 1. Detailed description of methods for genetic testing. Detailed description of methods for karyotyping, chromosomal microarray, exome sequencing and next generation sequencing. [file 12886_2021_2151_MOESM1_ESM.docx]

**Causes of poor eye contact in infants: A population-based study**

Mette Levinsen^1^, Malene Landbo Børresen^2^, Laura Roos^3^, Karen Grønskov^3^, Line Kessel^1,4^

^1^Department of Ophthalmology, Rigshospitalet, Glostrup, Denmark

^2^Department of Pediatrics and Adolescent Medicine, Rigshospitalet, Copenhagen, Denmark

^3^Department of Clinical Genetics, Rigshospitalet, Copenhagen, Denmark

^4^Department of Clinical Medicine, University of Copenhagen, Copenhagen, Denmark

**Detailed description of methods for genetic testing**

The main part of genetic investigations in the cohort were performed at the Department of Clinical genetics, Rigshospitalet. Karyotyping was performed by standard G-banding. Chromosomal microarray was performed with a 400K oligoarray (Agilent). Exome sequencing was performed with Human Core Exome Kit and sequencing on a NovaSeq platform with an average sequencing depth of 100X. Next generation sequencing (NGS) panels for the diagnoses albinism, retinal dystrophy, eye malformations and hearing impairment were analyzed using MiSeq Benchtop Sequencer (Illumina) and Sureselect Custom Region Enrichment (Agilent), and clinically significant sequence variants were confirmed with Sanger sequencing. Variants were evaluated according to ACMGs guidelines [1]. Three patients were analyzed at external laboratories: Two patients had NGS epilepsy panel performed, and one patient had sequencing and methylation analysis of MAGEL2 performed.

1. Richards S, Aziz N, Bale S, Bick D, Das S, Gastier-Foster J, et al. Standards and guidelines for the interpretation of sequence variants: a joint consensus recommendation of the American College of Medical Genetics and Genomics and the Association for Molecular Pathology. Genet Med. 2015;17:405–24.
