# Supplementary material for: Causes of poor eye contact in infants: a population-based study
Source: BMC Ophthalmol. 2021 Nov 7;21:388. doi: 10.1186/s12886-021-02151-7 (PMC8572507; doi:10.1186/s12886-021-02151-7)
Supplement: Supplementary file 2 — Additional file 2. Genetic findings in infants with poor eye contact. Detailed description of genetic findings among infants with poor eye contact. [file 12886_2021_2151_MOESM2_ESM.docx]

**Causes of poor eye contact in infants: A population-based study**

Mette Levinsen^1^, Malene Landbo Børresen^2^, Laura Roos^3^, Karen Grønskov^3^, Line Kessel^1,4^

^1^Department of Ophthalmology, Rigshospitalet, Glostrup, Denmark

^2^Department of Pediatrics and Adolescent Medicine, Rigshospitalet, Copenhagen, Denmark

^3^Department of Clinical Genetics, Rigshospitalet, Copenhagen, Denmark

^4^Department of Clinical Medicine, University of Copenhagen, Copenhagen, Denmark

**Genetic findings in infants with poor eye contact.**

| **Diagnosis** | **Gene** | **Inheritance** | **Mutation** | **pathogenicity** |
| --- | --- | --- | --- | --- |
| **Neurological abnormality** | | | | |
| Chromosomal abnormality |  |  | Karyotype: arr[GRCh37] 1q42.2q44(234030838_249212668)x3, 15q26.3(99902389_101151233)x3, 15q26.3(101196102_102465355)x1, reflecting an unbalanced reciprocal translocation t(1;15)(q42.2;q26.3), inheritance unknown |  |
| Chromosomal abnormality |  |  | Karyotype: arr[GRCh37] 1q43q44(242703697_249218792)x1, Xp22.33(169064_1322859)x3, reflecting an unbalanced form of a maternal reciprocal translocation t(1;X)(q43;p22.33). |  |
| Down syndrome (n=2) |  |  | Trisomy 21 |  |
| Coffin-Siris syndrome | *ARID1B*  (NM_001346813.1) | AD | c.4303C>T, p.(Gln1435*) | Pathogenic |
| COL4A1-associated porencefaly | *COL4A1*  (NM_001845.5) | AD | c.884_885del, p.(Lys295Argfs*11) | Likely pathogenic |
| Primary coenzyme 10 deficiency | *COQ4*  (NM_016035.4) | AR | c.23_33del, p.(Val8Alafs*19); c.532+6T>A, p.(splice variant) | Pathogenic |
| Epileptic encephalopathy | *CUX2*  (NM_015267.3) | AD | c.1768G>A; p.(Glu590Lys) | Pathogenic |
| Apert syndrome | *FGFR2*  (NM_000141.4) | AD | c.755C>G, p.(Ser252Trp) | Pathogenic |
| Omodysplasia | *GPC6*  (NM_005708.4) | AR | Intragenic, homozygous deletion in GPC6 | Pathogenic |
| Tay Sachs disease | *HEXA*  (NM_000520.5) | AR | Homozygous c.1100_1111del; p.(Gly367_Tyr370del) | Lijkely pathogenic |
| Schaaf-Yang | *MAGEL2*  (NM_019066.4) | AD/imprinting | c.1996dupC p.(Gln666Profs*47) | Pathogenic |
| Molybden co-factor synthesis deficiency | *MOCS2*  (NM_176806.3) | AR | Homozygous c.226G>A, p.(Gly76Arg) | VUS |
| Pitt-Hopkins syndrome | *TCF4*  (NM_001083962.1) | AD | c.1414del; p.(Val472Phefs*16) | Pathogenic |
| Cerebellar ataxia, mental retardation, and dysequilibrium syndrome | *WDR81*  (NM_001163809.1) | AR | Homozygous c.5335C>T, p.(Arg1779*) | Pathogenic |
| Salt and pepper regression syndrome | *ST3GAL5*  (NM_003896.4) | AR | Homozygous c.1060G>A, p.Asp354Asn | VUS |
| **Ocular disease** | | | | |
| Joubert syndrome | *NPHP1*  (NM_000272.3) | AR | Karyotype: arr[GRCh37] 2q13(110852960_110983703)x0  Homozygous 0,13Mb deletion including the entire *NPHP1* gene. | Pathogenic |
| Lebers congenital amaurosis | *RPE65*  (NM_0003296.2) | AR | Homozygous c.919_921del; p.(Ser307del) | Likely pathogenic |
| Oculocutaneous albinism | *TYR*  (NM_000372.4) | AR | c.1037G>A, p.(Gly346Glu); c.[575C>A;1185-6208A>G;1205G>A] | Likely pathogenic/pathogenic haplotype |
| Oculocutaneous albinism | *TYR*  (NM_000372.4) | AR | Homozygous c.1037G>A, p.(Gly346Glu) | Likely pathogenic |
| Oculocutaneous albinism | *TYR*  (NM_000372.4) | AR | Homozygous c.996G>A, p.(Met332Ile) | Pathogenic |
| Oculocutaneous albinism | *TYR*  (NM_000372.4) | AR | c.1118C>A, p.(Thr373Lys);c.1217C>T, p.(Pro406Leu) | Pathogenic/pathogenic |
| Oculocutaneous albinism | *TYR*  (NM_000372.4) | AR | c.1147G>A, p.(Asp383Asn), c.[575C>A;1185-6208A>G;1205G>A] | Pathogenic/pathogenic haplotype |
