# Supplementary material for: Causes of poor eye contact in infants: a population-based study
Source: BMC Ophthalmol. 2021 Nov 7;21:388. doi: 10.1186/s12886-021-02151-7 (PMC8572507; doi:10.1186/s12886-021-02151-7)
Supplement: Supplementary file 3 — Additional file 3. Electrophysiology testing including visual evoked potentials and electrode electroretinograms. Detailed description of electrophysiology testing among infants with poor eye contact. [file 12886_2021_2151_MOESM3_ESM.docx]

**Causes of poor eye contact in infants: A population-based study**

Mette Levinsen^1^, Malene Landbo Børresen^2^, Laura Roos^3^, Karen Grønskov^3^, Line Kessel^1,4^

^1^Department of Ophthalmology, Rigshospitalet, Glostrup, Denmark

^2^Department of Pediatrics and Adolescent Medicine, Rigshospitalet, Copenhagen, Denmark

^3^Department of Clinical Genetics, Rigshospitalet, Copenhagen, Denmark

^4^Department of Clinical Medicine, University of Copenhagen, Copenhagen, Denmark

|  | Patient 1 | Patient 2 | Patient 3 | Patient 4 | Patient 5 | Patient 6 | Patient 7 | Patient 8 | Patient 9 | Patient 10 | Patient 11 | Patient 12 | Patient 13 | Patient 14 | Patient 15 | Patient 16 |
| --- | --- | --- | --- | --- | --- | --- | --- | --- | --- | --- | --- | --- | --- | --- | --- | --- |
| Diagnosis | Idiopathic infantile nystagmus | Idiopathic infantile nystagmus | Idiopathic infantile nystagmus | Idiopathic infantile nystagmus | Epileptic encephalo-pathy | Hydroce-phalus | Cerebral  hemor-rhage | Pitt-Hopkins syndrome | Delayed  psycho-motor  development | Joubert syndrome | Oculo-motor  apraxia | Oculo-cutaneous albinism | Oculo-cutaneous albinism | Lebers congenital amaurosis | Isolated foveal hypoplasia | Microph-thalmia, cataract, retinal  detachment |
| Age at examination (months) | 3 | 6 | 5 | 12 | 5 | 9 | 10 | 6 | 9 | 20 | 5 | 7 | 6 | 7 | 13 | 5 |
| ERG | Normal | Normal | Normal | No signal | Normal | Normal | Normal | Normal | Normal | Normal | No signal | Normal | Normal | No signal | Normal | Uncertain signal |
| VEP | Normal | Normal | Normal | No signal | Abnormal | Abnormal | Abnormal | Normal | Normal | Normal | No signal | Misrouting | Misrouting | Normal | Normal | Uncertain signal |

**Electrophysiology testing including visual evoked potentials and electrode electroretinogram**
